# Memory for Object Location in Augmented Reality: The Role of Gender and the Relationship Among Spatial and Anxiety Outcomes

**DOI:** 10.3389/fnhum.2019.00113

**Published:** 2019-03-28

**Authors:** Francisco Munoz-Montoya, Camino Fidalgo, M.-Carmen Juan, Magdalena Mendez-Lopez

**Affiliations:** ^1^Instituto Universitario de Automática e Informática Industrial, Universitat Politècnica de València, Valencia, Spain; ^2^IIS Aragón, Departamento de Psicología y Sociología, Universidad de Zaragoza, Zaragoza, Spain

**Keywords:** augmented reality, spatial location, map pointing, anxiety, spatial strategy

## Abstract

The potential of augmented reality (AR) technology for the study of spatial memory and orientation is a new research field. AR defines systems that attempt to enhance the user’s experience with the physical world. In our app, we enhance the sense of sight by adding interactive 3D elements to the real environment. Our app can be used in any real environment so that the experimental conditions during the tasks and the way in which an individual navigates are similar to those used in real life. With AR, the experimenter has a high level of control of the task and can store the participant’s responses accurately. The classical factors that influence an individual’s performance on virtual spatial tasks are gender and cognitive factors. The influence of emotional factors on spatial performance has been studied more recently. Since AR tasks for the study of spatial memory and spatial orientation are new developments, little is known about the factors that are related to performance on tasks of this type. In our study, we tested 46 young adults (26 women) in an AR object-location task that was performed in a building. The participants had to memorize the position of eight virtual objects while they were walking through the environment. We also assessed the participants’ performance on an object-recall task, a map-pointing task, and a paper-and-pencil spatial orientation task. The self-reported importance of different spatial strategies for wayfinding and the levels of trait anxiety and wayfinding anxiety were also evaluated. Our findings indicate that men performed better on the spatial paper-and-pencil test and spent more time completing the learning phase of the AR task. The spatial memory for the location of the objects in AR and on the map correlated positively. Anxiety was related to individual differences in the self-reported use of a spatial orientation strategy, but the association among them was weak. Trait anxiety was positively related to the time employed by the participants during the learning phase of the AR task, whereas wayfinding anxiety correlated negatively with the preference for an orientation strategy. Our results highlight the importance of anxiety in spatial orientation.

## Introduction

The ability to maintain orientation within the spatial environment is one of the most fundamental cognitive functions in humans. In fact, spatial orientation is involved in everyday tasks such as finding the exit in an unknown building or finding one’s way in a complex environment. Given that spatial orientation includes multiple and complex cognitive processes (Wolbers and Hegarty, 2010), it is not surprising that individuals differ in their ability to orientate themselves in space, ranging from individuals who get lost easily to those with excellent orientation skills.

Most of the studies that examine factors influencing spatial navigation have focused on cognitive and biological variables (Siegel and White, 1975; Lawton, 1994; Lawton, 1996; Piccardi et al., 2011a). One approach for examining individual differences based on cognitive variables is to investigate which strategy people use in spatial orientation. As individuals may analyze spatial information differently, they may therefore employ different strategies to find a destination (Lawton, 1996). Three spatial strategies or cognitive styles have been described based on the information people seek in order to orientate themselves in an environment: landmark, route, or survey strategies (Siegel and White, 1975). Landmark and route strategies are based on an egocentric reference frame and are less sophisticated than the survey strategy. The landmark strategy is based on perceptually salient or important cues for individuals, whereas the route cognitive style uses paths that are connected to different landmarks (Siegel and White, 1975). In contrast, an extrinsic reference frame is used in the survey strategy in which people use a cognitive map to orientate themselves in an environment (Pazzaglia and De Beni, 2001). Similar to the cognitive styles described above, other authors proposed different wayfinding strategies that people use to orientate themselves indoors in the Indoor Wayfinding Strategy Scale (Lawton,1996). The orientation strategy is similar to the survey strategy, the indoor route strategy is similar to the outdoor route strategy, and the building configuration strategy refers to symmetry in the building and corridor angles. In our experiment, the AR spatial task was performed inside a building; therefore, we investigated the self-reported strategies preferred by participants indoors (orientation, route, and building configuration) using the Indoor Wayfinding Strategy Scale (Lawton, 1996) instead of the outdoor orientation strategies (landmark, route, or survey strategies). However, it should be noted that there is a tendency to use similar wayfinding strategies indoors and outdoors (Lawton, 1996). Thus, a person who prefers a survey strategy outdoors will prefer an orientation strategy indoors.

Another variable that may influence spatial orientation is gender. Gender differences in spatial orientation have been described by several authors (Coluccia and Louse, 2004; Iachini et al., 2005; Piccardi et al., 2011b; León et al., 2016) and could depend on various factors, such as the difficulty of the task, emotional factors, or the spatial strategy used. Accordingly, it has been argued that men and women seem to use different strategies to orient themselves in space. Men are prone to use survey orientation more than women, which is usually more efficient than landmark or route strategies (Lawton, 1994, 1996), whereas women are reported to use route strategies (Lawton, 1996; Lawton and Kallai, 2002). Despite the fact that some studies show that men outperform women in spatial orientation, the results are conflicting and there was also an absence of sexual dimorphism in spatial orientation in other studies (see Coluccia and Louse, 2004 for review).

One likely reason for the discrepancies between studies is that other variables, such as personality, may influence performance on spatial tasks (Coluccia and Louse, 2004). For example, high scores in psychoticism and neuroticism are associated with a poorer spatial performance (Burles et al., 2014; Walkowiak et al., 2015). Anxiety has also been observed to predict weaker performance on some spatial reasoning tasks (Lawton, 1996; Schmitz, 1997). Mueller et al. (2009) found that children with anxiety disorder exhibit overall impaired performance in a virtual version of the Morris Water Maze when compared to a control group.

The role of wayfinding anxiety in spatial orientation has received more attention. Wayfinding anxiety refers to anxiety about performing spatial tasks (Lawton, 1994) and is also related to the fear of getting lost (Schug, 2016). Wayfinding anxiety has been associated with poorer performance, lower self-efficacy, and less pleasure in exploring in spatial tasks (Lawton, 1994; Coluccia and Louse, 2004; Pazzaglia et al., 2018). Interestingly, even though women have been described as being more anxious than men when wayfinding (Lawton, 1994, 1996; Schmitz, 1997; Schug, 2016) and having less self-confidence to solve spatial tasks (Picucci et al., 2011; Nori and Piccardi, 2015), they perform comparably and achieve similar results as men in spatial tasks such as mental rotation tasks (Neuburger et al., 2012), wayfinding tasks (Lawton, 1996), and spatial environmental tasks (Nori and Piccardi, 2015).

Exploring the possible influence of individual differences in spatial orientation can be assessed using several spatial tests such as self-report questionnaires, paper-and-pencil psychometric tests, bi-dimensional maps, or by means of environmental tasks. In the self-report questionnaires, people assess their own orientation skills and strategies (Lawton, 1996; Claessen et al., 2016). Paper-and-pencil tests assess spatial perception, spatial visualization, and mental rotation (Mitolo et al., 2015). In bi-dimensional maps, all the spatial information is available from a single point of view. In tasks using bi-dimensional maps, the participants do not move around in the environment, but instead make a mental representation of it (Castelli et al., 2008). On another hand, in environmental tasks, an individual’s performance can be evaluated in real (Labate et al., 2014) or virtual reality (VR) environments (Walkowiak et al., 2015; León et al., 2016; Rodríguez-Andrés et al., 2016; Cárdenas-Delgado et al., 2017).

VR environments meet the needs of several research domains that are related to spatial cognition and navigation (Bohil et al., 2011; León et al., 2016). VR has been seen to be a valid and feasible tool for investigating spatial memory, with advantages in terms of methodological issues (Rodriguez-Andres et al., 2018). VR allows participants to be exposed to complex and natural-appearing environments. In contrast to real-world navigation experiments, which are difficult to control and execute, VR facilitates the control of delivered stimuli, the manipulation of variables, and the recording of measurements.

Interestingly, VR has also been used to investigate the effect of emotional variables in the spatial orientation both of adults and children (Burles et al., 2014; Walkowiak et al., 2015; Zlomuzica et al., 2016; Pazzaglia et al., 2018; Rodriguez-Andres et al., 2018). Specifically, it has been shown that the induction of an anxious emotional state decreased spatial context retrieval in healthy participants (Zlomuzica et al., 2016). A poorer spatial performance in VR spatial tasks has been described in healthy participants with higher scores in neuroticism (Burles et al., 2014) and psychoticism (Walkowiak et al., 2015). In children, withdrawal behaviors have been related to an increase in exploratory behavior in a VR spatial orientation task (Rodriguez-Andres et al., 2018). Despite the fact that VR has been used to investigate spatial orientation in several studies, a remaining restriction of this technology is cybersickness. This side effect sometimes leads to nausea, vertigo, and vomiting, limiting the widespread adoption of VR for therapeutic or training applications requiring repeated use over time.

Augmented reality (AR) is a technology that allows the experimenter to superimpose objects upon the real world in order to supplement reality. AR can be used in any real environment so that the experimental conditions during the tasks and the way in which participants navigate are similar to those in real life. Like VR, AR allows the control of the variables of the task and the storage of the participant’s responses (Juan et al., 2014) but without the limitation of inducing cybersickness. Despite the fact that AR has great potential to assess cognitive processes, to our knowledge, only two studies (Juan et al., 2014; Mendez-Lopez et al., 2016) have used AR to evaluate spatial ability while a person is moving, showing promising results.

The above-mentioned studies point out the existence of multiple spatial tests to assess how individual differences might influence spatial orientation. However, it should been taken into consideration that the spatial information available in each test and the way in which the task can be solved is different in self-report questionnaires, paper-and-pencil psychometric tests, bi-dimensional maps, or environmental tasks. Accordingly, in bi-dimensional maps, all the spatial information is available from a single point of view, whereas in real or virtual environments, participants move around in the environment. In fact, in real or virtual indoor environments, participants can orientate themselves using orientation, route or building configuration strategies, whereas a survey strategy is needed to solve a bi-dimensional map task. On another hand, basic spatial abilities such as spatial perception or mental rotation are assessed by psychometric tests. Therefore, more studies are needed in order to further investigate the relationship between psychometric tests, bi-dimensional map tasks, and large-scale spatial tasks.

However, discrepant results about the influence of gender in spatial orientation have also been described. Moreover, the relationship of anxiety and spatial skills of individuals and their performance on a spatial orientation task in a complex real-world setting using an app based on AR technology has not yet been investigated.

Accordingly, the research questions are: (1) Does gender has any effect on spatial performance (i.e., the AR task for object location, the bi-dimensional map-pointing task, the spatial orientation test, and self-reported spatial strategy of preference) and on anxiety (i.e., wayfinding anxiety and trait anxiety)? (2) Is there any significant relationship among participants’ performance, regardless of gender, on the environmental AR task, the map-pointing task, the paper-and-pencil spatial orientation test, the preferred spatial strategy, and anxiety? (3) Could gender influence any possible significant associations that may be found between participants’ spatial performance and anxiety outcomes?

## Materials and Methods

### Participants

The participants included 46 adults (78.3% undergraduates and 21.7% graduates). The gender distribution was 43.5% men and 56.5% women. The men’s mean age was 24.65 ± 8.54 years. In the case of women, the mean age was 23.73 ± 7.71 years. They were recruited at the University of Zaragoza, Teruel Campus, through campus advertising. In the advertisement, potential participants were encouraged to learn more about their spatial ability. Each participant received a report describing his/her results on the tests of the study as a reward. The final sample was selected after applying the inclusion criteria to a larger sample composed of 105 adults. The participants were right-handed, did not have any motor or sensory impairment, had not suffered a brain injury, were not treated with a medication that could potentially impair their cognitive functioning, and all participants frequented the building where the study took place weekly at least three days a week. Table 1 summarizes the main characteristics of the final sample. We determined the town where the participants grew up and their wayfinding experience at ages 3-15 years using the scale reported by Schug (2016). The scale asked the participants how far from home in km they were allowed to go without an adult (by themselves or with friends) at the following ages: 3-4 years old, 5-7 years old, 8-10 years old, 11-13 years old, and 13-15 years old. We also determined the participants’ experience playing with smartphones or AR apps (Table 1). The participants gave written informed consent prior to the study. The Ethics Committee of the Universitat Politècnica de València, Spain, approved the study. The study was conducted in accordance with the declaration of Helsinki.

**Table 1:** Characteristics of the sample.

Measure	*Men (n = 20)*	*Women (n = 26)*
		
*Age (years) M (SD)*	24.65 (8.54)	23.73 (7.71)
**Student status**		
Undergraduate	80%	76.9%
Graduate	20%	23.1%
**Childhood wayfinding experience**		
Town (%urban/%rural)	90% / 10%	92.3% / 7.7%
Score *M (SD)*	15.65 (4.27)	15.85 (3.96)
**Experience (%)**		
**Using mobiles for playing**		
Never	25%	23.1%
Once a month	20%	19.2%
Once a week	25%	23.1%
Almost daily	15%	15.4%
Every day	15% ( < 1 h)	19.2% ( < 1 h)
**Playing AR apps**	65% (hardly ever)	80% (hardly ever)


*The town where the participants grew up was coded as *urban* if population ≥ 2,500 and *rural* if population < 2,500. The participants who played using smartphones every day were given the following answer options: *“ < 1 h a day”, “2-3 h a day”* and *“ > 4 h a day”*. They played less than 1 h in all the cases. The question to determine the participants’ experience playing AR apps consisted of three answer options: *“never”, “sometimes”* and *“often”*. No participant responded *“often”.* All participants frequented the building in which the study took place, at least, three days a week (item scored on a 1-5 Likert scale; from *“1. At least one day a week”* to *“5. At least five days a week”*).*

### The AR Task

The task consists of a short-term memory test for object location, which is performed using an AR app that is played on a smartphone (Lenovo Phab 2 Pro) (Munoz-Montoya et al., 2019). The app allows the participants to tour a real room/building. The participant must look for virtual objects which are located in the real building and remember their locations in order to place them in their correct real-world locations later.

For this purpose, the examiner first configures the environment for the task in two phases: 1) Environment scanning. The scanning of the environment only has to be done once. In this phase, the examiner scans the real environment where the study will be carried out. 2) Object configuration. The examiner places the different 3D objects in the real scanned environment.

**FIGURE 1:**
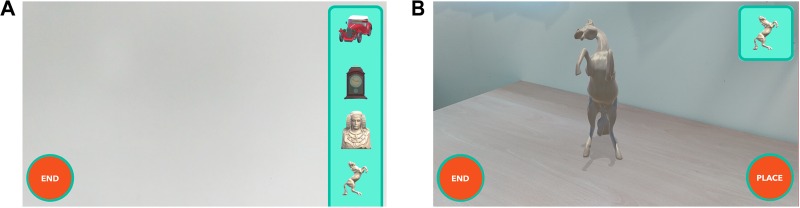
Testing phase. **(A)** Object selection. The sidebar on the right side shows the images of the objects to be placed in the real environment. **(B)** Object positioning. The sculpture is placed on the correct table.

For the participants, there are two phases: the learning phase and the testing phase. In the learning phase, the participants are asked to inspect the scanned environment, looking for virtual objects, and to remember their locations. There are eight objects. In the testing phase, the examiner asks the participants to place the objects in the correct location using the app. For this purpose, a list of objects that the participants have to find is shown on the right side of the screen (Figure 1A). The participants have to select these objects one by one to place them in the environment. Once the participants have selected an object, they have to place it in the real environment. In order to achieve this, the device has to be moved to the desired location and focused with the camera on the precise place. The virtual object is shown in the center of the screen and is adapted to the flat surface of the environment (Figure 1B). Once the object is displayed on the desired surface, the participant presses the “place” button. If this position is correct, the user is informed about the success, the object is anchored in that place and disappears from the list of available objects. The user can then continue positioning the rest of the objects. In contrast, if it is a failure, the participant is informed about the remaining available attempts. This object still appears on the list of available objects as long as the total number of attempts has not been achieved. More details about the task can be found in Munoz-Montoya et al. (2019).

An examiner accompanied the participant during the task. Before starting to use the app, the examiner told the participant about the goal of the task. The examiner showed the participant how to hold the device, how to move around in the virtual environment and how the app worked.

### The Phases

The AR task comprised two phases: the learning phase and the testing phase. In the learning phase, the participants were asked to inspect two floors of a familiar building of the campus, looking for virtual objects, and to remember their locations, without time limitations. Time limit was not established in order to avoid time-pressure, which could be an additional source of stress. There were eight objects. Their distribution in the building was the same for all participants. In section “The Environment,” we describe the route for the inspection, the objects, and their location. The participants did not receive any help regarding where the objects were, the objects that they had seen, or those that they were required to see. The examiner accompanied each participant during this phase indicating the route to follow for the inspection. All of the participants did the same tour and, consequently, looked for the objects in the same order.

Once a participant had inspected the last object, the learning phase ended. Then, the experimenter accompanied the participant to the beginning of the route and the testing phase started. The time between phases was 3 min because this was the time needed to go from the place where the learning phase finished to the place where the testing phase began.

In the testing phase, the examiner asked the participants to place the objects in the correct location using the app, without time limitations. The participants were told beforehand that they were allowed three attempts to locate each object. The examiner showed the participants how this phase worked. The participants were informed that their success in this phase was determined by the correct location of the objects, regardless of the route followed for this purpose and the order in which they located the objects. The participants were also informed that, at any time, they could select a different object for placing.

In our study, we considered four variables related to performance on the AR task: the time spent on the learning phase (in seconds), the time spent on the testing phase (in seconds); the number of objects that were located correctly during the testing phase (LocObj); and the number of errors committed during the testing phase (ErrObj).

### The Instrumentation

The AR app was played using a Tango smartphone, Lenovo Phab 2 Pro (size: 6.4 inches; weight: 259 grams). The participants held the smartphone using an external case to make handling easy. The orientation of the screen was *landscape*.

### The Environment

**FIGURE 2:**
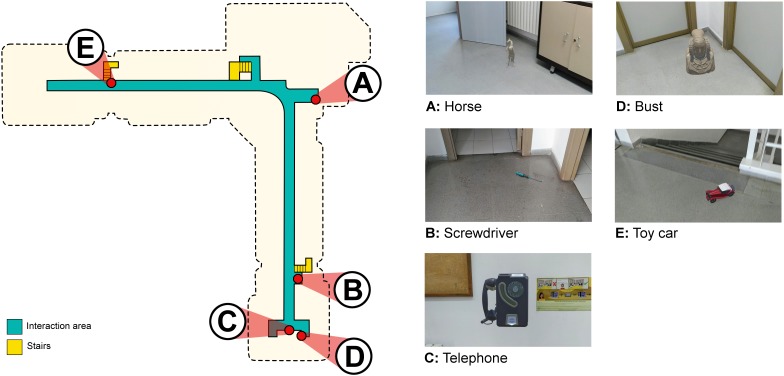
A schematic top view of the interaction area and the location of the five virtual objects on the second floor. The dashed black line shows the shape of the building. The objects are designated by letters in order of appearance during the learning phase: **(A)** horse; **(B)** screwdriver; **(C)** telephone; **(D)** bust; **(E)** toy car. On the right side of the figure is an image of where the objects were located during the phase.

**FIGURE 3:**
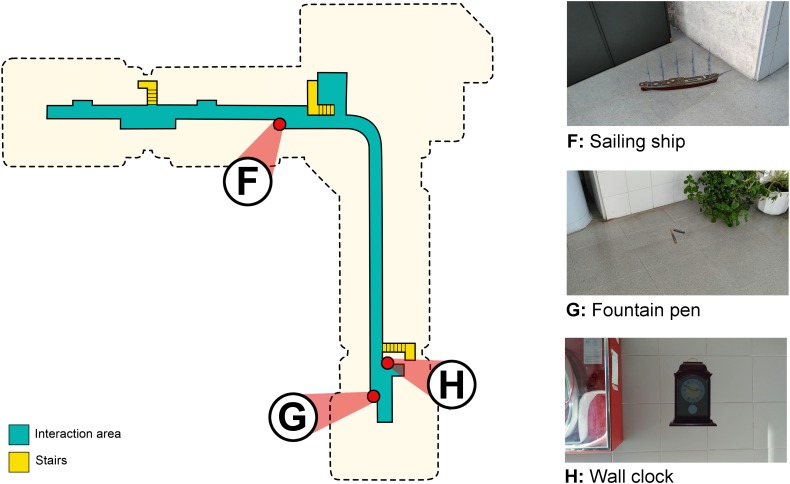
A schematic top view of the interaction area and the location of the three virtual objects on the first floor. The objects are designated by letters in order of appearance during the learning phase: **(F)** sailing ship; **(G)** fountain pen; **(H)** wall clock. The dashed black line shows the shape of the building. On the right side of the figure is an image of the objects as they appeared on the phase.

The task was carried out in the communal areas of the first and second floor of the School of Social and Human Sciences of the University of Zaragoza. The areas to be explored during the task consisted of 282 square meters on the first floor and 331 square meters on the second floor. Figure 2 shows the shape of the environment on the second floor and the location of the virtual objects. Figure 3 shows the same aspects corresponding to the first floor. There were five objects on the second floor and three objects on the first floor. The objects were decorative items (i.e., bust, horse, toy car, sailing ship, and wall clock) or everyday objects (i.e., telephone, fountain pen, and screwdriver). The location of the objects was decided based on the length and shape of the areas for exploration and their visual cues. The objects were placed on a wall (i.e., wall clock and telephone), the floor (i.e., bust, toy car, sailing ship, fountain pen, and screwdriver) and a bookcase (i.e., horse). Their location was proximal to existing cues (i.e., bookcase, doors, stairs, plants, recycling point, fire hose, fire extinguisher, and board). In addition, the fact that there were physical cues in the real environment guaranteed an optimal recognition of the environment. The app locates the mobile device through the recognition of distinctive visual points. An environment composed of uniform places or identical rooms without distinguishing elements would increase the difficulty in its correct recognition. Thanks to these cues, the app correctly recognizes the environment and facilitates the precise placement of the augmented objects.

**FIGURE 4:**
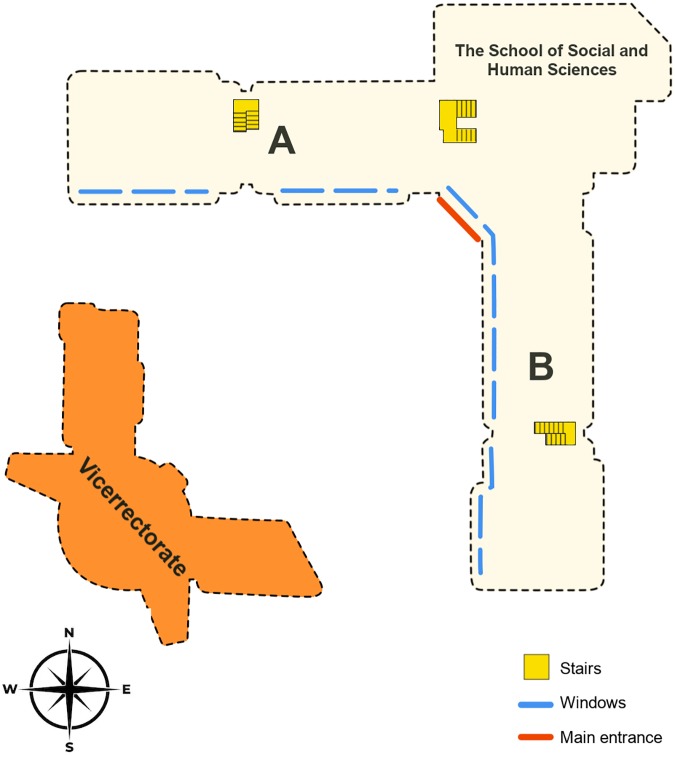
A schematic top view of the cardinal orientation of the School of Social and Human Sciences and surrounding buildings. The School consists of an L-shape building. From the main entrance (in the angle), part **(A)** is in the north of the building and part **(B)** is in the east of the building. Dotted blue lines represent the position of the windows in the building.

Together with proximal cues, distal cues were also available: the main entrance of the building, the windows, the natural sunlight, and the Vicerrectorate building. The School of Social and Human Sciences is an L-shaped building, the main entrance is at the angle and faces southwest. From the main entrance, one part of the building (A) is in the north and the other in the east (B) (see Figure 4). The entrance is an open area without walls, so it can be observed both from the first and the second floor. The Vicerrectorate building is located in the southwest and can be also observed both from the first and the second floor of the School of Social and Human Sciences, due to the fact that all the windows are located in the southern (part A) and the western face of the building (part B). Therefore, natural light enters the building only through one face of the building. All stairs are placed in the north-eastern face of the building, where there are no windows.

**FIGURE 5:**
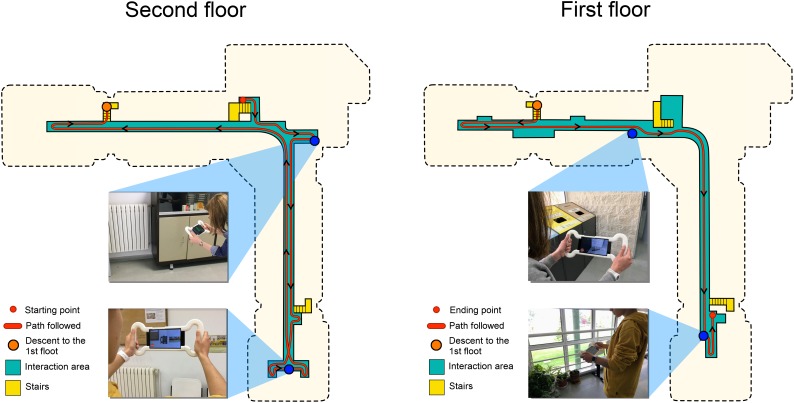
The route made by the participants in the learning phase and location of the objects.

Figure 5 shows the route followed by the participants in the learning phase during the tour directed by the examiner. The tour involved exploring hallways and corridors where there were no objects. No objects were placed on the stairs for safety reasons. The examiner warned each participant to look at the floor when going down the stairs.

### The Object-Recall Task

The object-recall task consisted of a free recall of the objects inspected in the AR task. The participants answered verbally, and the examiner wrote down their answers without giving any feedback. The examiner asked each participant “What objects have you inspected with the AR app? Please, list all you remember”. There was no time limit for the participant’s response, but we established an internal time limit of three minutes to avoid unnecessary response delay. We considered two variables related to performance on the object-recall task: the percentage of errors committed (%ErrRecall), and the percentage of omissions made (%OmitRecall).

### The Map-Pointing Task

**FIGURE 6:**
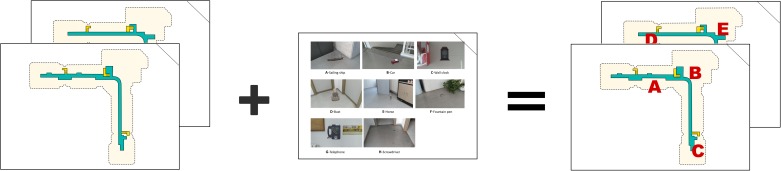
The tools used in the map-pointing task.

In this task, the participants viewed two empty maps, which corresponded to the first and the second floors of the building. Each map was a two-dimensional simplified map in which hallways, stairs, corridors, and rooms were illustrated. The floor of each map was indicated in print and orally. No other visual cues were shown. The participants also viewed a composite of the eight objects of the AR task labeled with letters. The maps and the composite with the objects were printed on a sheet of DIN A-4 sized paper. Figure 6 shows these tools. The examiner asked each participant to point to each object in its correct location according to the AR task, writing its letter on the correct map. There was no time limit to accomplish the task. The performance scores on the map-pointing task were the time spent to complete the task in seconds (ObjPoint) and the percentage of objects correctly located (%ObjPoint). We considered that an object was located correctly on the map when it was pointed to within a 3 mm radius of its precise location. Pointing accuracy is of interest to us because it seems to be related to orientation strategies and spatial anxiety (Bryant, 1982; Lawton, 1996).

### The Spatial Orientation Test

The participants completed a paper-and-pencil spatial orientation test. They performed the Perspective Taking/Spatial Orientation Test (PTSOT; the revised version of Hegarty and Waller, 2004) from the test used by Kozhevnikov and Hegarty (2001), following the procedures indicated by the authors. This test consists of 12 items that assess the participant’s ability to orientate spatially and to image different perspectives. Five minutes were given to perform this test. We considered two variables related to performance on the PTSOT: the total score (PTSOTsc) and the percentage of unattempted items (%uPTSOT).

### Self-Reported Strategies

The participants completed a self-report questionnaire about their strategies for wayfinding in unfamiliar buildings (the translated version of the Indoor Wayfinding Strategy Scale used by Lawton, 1996). This questionnaire is composed of 13 items. The items were statements about certain behaviors that could emerge or certain information that could be used for spatial orientation in a building or a large complex. The participants rated the probability/importance of the statements on a five-point Likert scale. With the questionnaire, we measured the degree of importance of three different strategies during wayfinding: the building configuration strategy (BuilConf), the route strategy, and the orientation strategy. The BuilConf strategy consisted of the importance attached to a uniform layout of the building or complex. The route strategy is based on information about the route to be followed (i.e., visual cues or directions from another person). The orientation strategy is based on directional cues.

### Anxiety Scales

The level of trait anxiety was measured using the 20 items of the State-Trait Anxiety Inventory validated in Spain (STAI; Guillén-Riquelme and Buela-Casal, 2011, from the original version of Spielberger et al., 1970). The STAI items were scored on a four-point Likert scale. The raw scores of trait anxiety were used (TraitAnx). The eight items of the Wayfinding Questionnaire (Claessen et al., 2016) were also used. This questionnaire measures self-reported spatial anxiety when navigating in unfamiliar places. The items are scored on a 1-7 Likert scale. We used the raw scores of the wayfinding anxiety factor of the questionnaire (WayAnx).

### Procedure

First, the participants filled out a questionnaire on their own on the Internet between 5 and 15 days prior to performing the spatial tests. The questionnaire was created using Google Forms. The link to the questionnaire was distributed through their personal e-mail with a personal code to maintain anonymity. The questionnaire included items related to the participants’ general characteristics (i.e., age, educational level, familiarity with the environment, childhood wayfinding experience (Schug, 2016), and experience playing with smartphones or AR apps; see Table 1 for more information) and the selected items of the self-report questionnaires in the following order: wayfinding anxiety of the Wayfinding Questionnaire (Claessen et al., 2016), Childhood Wayfinding Experience Scale (Schug, 2016), Indoor Wayfinding Strategy Scale (Lawton, 1996) and trait anxiety of the STAI (Guillén-Riquelme and Buela-Casal, 2011). Afterward, we invited the participants to complete the spatial tests individually using the following established sequence: AR task (25–30 min.), PTSOT (5 min.), object-recall task (1–2 min.), and map-pointing task (5–7 min.). No time limit was established for any of the tasks except for the PTSOT. We conducted the testing in the School of Social and Human Sciences of the University of Zaragoza. The testing took place from Monday to Friday between 9:00 A.M. and 19:00 P.M.

### Data Analysis

The Kolmogorov-Smirnov test was used to check the normal distribution of the dataset variables. Only the Route strategy variable and the ErrObj variable of the AR task followed a normal distribution. Nonparametric tests, which are more suitable for distributions of this type, were used with the entire data-set.

For the research question (1), Mann-Whitney *U*-tests were applied to investigate gender differences in the variables related to the spatial tasks: the AR task [LocObj, ErrObj, Learn (s.) and Test (s.)]; the Object-recall task (%ErrRecall, %OmitRecall); the Map-pointing task [%ObjPoint and ObjPoint(s.)]; and the PTSOT (PTSOTsc, %uPTSOT). The same statistical analysis was used to study gender differences in self-reported wayfinding strategies (BuilConf, Route and Orientation) and in anxiety outcomes [trait anxiety (TraitAnx) and wayfinding anxiety (WayAnx)]. For the research question (2), partial Spearman correlations, extracting the influence of gender, were calculated.

For the research question (3), in order to study the relationship between wayfinding anxiety and variables related to the spatial navigation performance (i.e., AR task and self reported wayfinding strategies) as mediated by Gender, Spearman’s correlations were calculated separately for each gender, considering these variables, and the significant correlation coefficients were compared using Fisher’s Z-test (Hidalgo et al., 2014). All of the analyses were conducted using IBMSPSS Statistics, version 19.0. The results were considered to be statistically significant if *p* < 0.05.

## Results

Table 1 shows descriptive statistics for the four variables related to the general characteristics of the sample: age, student status, childhood wayfinding experience, and percentage of experience using smartphones or AR apps to play. In the case of childhood experience, we present the descriptive statistics for the percentage of men and women who grew up in an urban or a rural environment. The mean of the total score for each gender is also shown. The mean of this score was the sum of the distance in km reported in each age range on the scale (min.-max. = 5-30). The scores of the males and females revealed that the wayfinding experience was similar between genders (Table 1).

Table 2 shows the results of the statistical analysis. The Mann-Whitney *U*-test revealed statistical differences between men and women for the time spent on the learning phase of the AR task (*U* = 171, *Z* = -2.0, *p* = 0.049, *r* = -0.29) and for the PTSOT score (*U* = 156, *Z* = -2.3, *p* = 0.021, *r* = -0.34). The men required more time in the learning phase of the AR task. Also, the men showed better performance on the PTSOT. A lower score on the PTSOT reflects a good performance on the test. The rest of the studied variables did not reveal significant differences.

**Table 2:** Mean scores (standard deviations) and Mann-Whitney’s *U* tests for the variables used in the study (*N* = 46).

Measure	*Men*	*Women*	*U-Test*	*Sig.*
				
	*M (SD)*	*M (SD)*	*U,(Z)*	*P, (r, if applicable)*
**AR task**				
LocObj	5.65 (1.31)	5.77 (1.81)	232, (0.6)	0.532
ErrObj	7.85 (4.32)	7.88 (5.49)	248, (-0.2)	0.798
Learn (s.)	590.67 (90.88)	528.70 (128.83)	171, (-2.0)	0.049, r = -0.29
Test (s.)	559.11 (214.87)	565.73 (181.57)	249, (-0.2)	0.807
**Object recall task**				
%ErrRecall	0.62 (2.79)	0.96 (3.40)	253, -0.4	0.717
%OmitRecall	5.00 (9.42)	2.40 (5.02)	237, -0.7	0.488
**Map reading task**				
ObjPoint(s.)	143.50 (48.28)	179.62 (90.61)	206, -1.2	0.231
%ObjPoint	63.12 (31.01)	59.13 (25.39)	221, -0.9	0.381
**Spatial orientation**				
PTSOTsc	31.24 (26.37)	52.65 (39.38)	156, -2.3	0.021, r = -0.34
%uPTSOT	24.17 (25.92)	23.72 (21.30)	256, -0.1	0.928
**Self-reported strategies**				
BuilConf	7.35 (2.54)	7.88 (2.55)	241, -0.4	0.670
Route	15.55 (2.84)	16.12 (2.30)	225, -0.8	0.441
Orientation	16.70 (4.86)	14.62 (2.71)	188, -1.6	0.111
**Anxiety outcomes**				
TraitAnx	24.75 (11.33)	26.19 (9.81)	237, -0.5	0.618
WayAnx	25.90 (9.92)	30.50 (10.24)	200, -1.3	0.187


*The performance scores on the AR task: LocObj = number of objects located; ErrObj = number of errors made; Learn (s.) = time spent on the learning phase; Test (s.) = time spent on the testing phase. The performance scores on the objects-recall task: %ErrRecall = percentage of errors; %OmitRecall = percentage of objects omitted. The performance scores on the map-pointing task: ObjPoint(s.) = time spent indicating the objects; %ObjPoint = percentage of objects successfully indicated. PTSOTsc = score; %uPTSOT = percentage of unattempted items. BuilConf = building configuration strategy. TraitAnx = trait anxiety. WayAnx = wayfinding anxiety.*

Table 3 shows the correlation among the variables studied. Significant correlations were found between variables of the performance in the AR spatial task. Specifically, the number of objects located correctly in the AR task correlated negatively with the number of errors committed (*r* = -0.97, *p* < 0.001). The time spent to complete the learning phase correlated positively with the number of objects located correctly in the task (*r* = 0.33, *p* = 0.024) and negatively with the number of errors committed (*r* = -0.33, *p* = 0.025). We also found that participants who committed more errors took more time to complete the testing phase of the AR task (*r* = 0.33, *p* = 0.028).

**Table 3:** Partial Spearman correlations (*N* = 46).

	2	3	4	5	6	7	8	9	10	11	12	13	14	15
(1) LocObj	-0.97^∗∗^	0.33^∗^	-0.29	-0.01	-0.46^∗∗^	-0.13	0.61^∗∗^	0.001	0.12	0.22	0.07	0.07	0.06	-0.02
(2) ErrObj		-0.33^∗^	0.33^∗^	0.02	0.48^∗∗^	0.13	-0.65^∗∗^	-0.02	-0.12	-0.23	-0.06	-0.10	0.05	0.06
(3) Learn(s.)			0.05	-0.002	-0.09	-0.12	0.25	-0.12	0.05	0.07	0.05	0.19	0.39^∗∗^	0.05
(4) Test(s.)				0.08	0.03	0.27	-0.27	0.03	0.10	-0.33^∗^	0.07	0.22	0.05	-0.04
(5) %ErrRecall					0.03	0.11	-0.14	0.002	-0.05	0.03	0.03	0.24	-0.02	-0.21
(6) %OmitRecall						0.09	-0.40^∗^	-0.07	-0.13	0.09	0.06	-0.36^∗^	0.14	0.19
(7) ObjPoint(s.)							-0.27	-0.01	0.31^∗^	0.04	-0.15	-0.07	-0.18	-0.03
(8) %ObjPoint								0.07	0.05	0.33^∗^	0.16	0.11	0.01	0.06
(9) PTSOTsc									0.15	-0.17	0.19	0.08	-0.18	-0.12
(10) %uPTSOT										-0.002	0.38^∗^	0.000	-0.12	-0.06
(11) BuilConf											0.12	-0.09	0.09	0.07
(12) Route												0.03	-0.08	0.01
(13) Orientation													–009	-0.32^∗^
(14) TraitAnx														0.33^∗^
(15) WayAnx														1.00


*The performance scores on the AR task: LocObj = number of objects located; ErrObj = number of errors made; Learn (s.) = time spent on the learning phase; Test (s.) = time spent on the testing phase. The performance scores on the objects-recall task: %ErrRecall = percentage of errors; %OmitRecall = percentage of objects omitted. The performance scores on the map-pointing task: ObjPoint(s.) = time spent indicating the objects; %ObjPoint = percentage of objects successfully indicated. PTSOTsc = score; %uPTSOT = percentage of unattempted items. BuilConf = building configuration strategy. TraitAnx = trait anxiety. WayAnx = Wayfinding anxiety. ^∗^*p* < 0.05; ^∗∗^*p* = 0.001. Size of effect: *r* = 0.3-0.5 medium; *r* > 0.5 = large (Cohen, 1988).*

Regarding the correlations between wayfinding anxiety and variables related to the spatial performance as mediated by Gender, there was a significant negative correlation between wayfinding anxiety and the orientation strategy in men (*r* = -0.45; *p* = 0.047), but this correlation was not significant in women (*r* = -0.13; *p* = 0.54). Significance testing using Fisher’s *Z*-test revealed no significant differences between men and women in this association (*z* = 1.11, *p* = 0.28).

On another hand, Spearman’s partial correlations controlling for gender differences revealed an association between the AR task and the object-recall task. The percentage of objects omitted in the recall task correlated negatively with the number of objects located correctly in the AR task (*r* = -0.46, *p* = 0.001) and positively with the number of errors committed in the AR task (*r* = 0.48, *p* = 0.001). Interestingly, the more objects correctly located in the AR task, the more the objects correctly pointed to in the map task (*r* = 0.61, *p* < 0.001). As expected, a negative correlation was found between the number of errors in the AR task and the percentage of objects that were pointed to correctly in the map task (*r* = -0.65, *p* < 0.001). It was also observed that the more objects omitted in the recall task, the fewer the objects correctly pointed to in the map task (*r* = -0.40, *p* = 0.006). In addition, a positive correlation was found between the percentage of unattempted items in the spatial orientation paper-and-pencil PTSOT test and the time spent to complete the map-pointing task (*r* = 0.31, *p* = 0.041).

In relation to the self-report indoor wayfinding strategies, the level of importance of the building configuration for wayfinding correlated positively with the percentage of objects pointed to correctly on the map (*r* = 0.33, *p* = 0.025) and negatively with the time spent to complete the testing phase of the AR task (*r* = -0.33, *p* = 0.028). On another hand, a positive correlation was found between the level of importance of the route strategy and the unattempted items on the PTSOT (*r* = 0.38, *p* = 0.011). Interestingly, the more importance attached to the orientation strategy, the fewer the objects omitted during the recall task (*r* = -0.36, *p* = 0.016).

Finally, with regard to the anxiety outcomes, a positive correlation was found between trait anxiety and wayfinding anxiety (*r* = 0.33, *p* = 0.028). Also, the higher the participant’s level of trait anxiety, the more the time he/she spent on the learning phase of the AR task (*r* = 0.39, *p* = 0.007). In addition, the greater the preference for directional cues for orientation (i.e., orientation strategy), the lower the participant’s level of wayfinding anxiety (*r* = -0.32, *p* = 0.033).

## Discussion

We studied the influence of gender in cognitive (self-reported spatial strategies) and anxiety outcomes as well as in the performance of three spatial tasks (a real-world orientation task using AR, a map-pointing task, and a paper-and-pencil spatial orientation task), which required different spatial orientation strategies/spatial abilities to be solved (route and orientation spatial strategies and perspective-taking spatial ability, respectively). The possible relations between the studied factors were also analyzed.

To our knowledge, this is the first study that investigates the role of gender in the performance of an object-location task using AR in a real-world setting and the relationship between this performance and cognitive factors and anxiety. Our results show no differences between men and women, either in the performance of the AR task for object-location (i.e., objects located and errors made) or in the map-pointing task or the recall task. However, men outperformed women in the paper-and-pencil psychometric test of spatial orientation (i.e., PTSOT).

The AR task was performed in a two-floor building. Although virtual objects were placed in specific areas close to proximal cues to facilitate their correct location in the testing phase, the task was not easy for the participants. In fact, the mean of correctly located objects out of 8 objects was: 5.6 for men and 5.7 for women, and the number of errors was almost 8 (for men and women). Therefore, nobody was able to locate all the 8 objects correctly without making errors. The cues available in the environment and, specially, those proximal to the virtual objects, could promote the use of a spatial strategy in the participants based on route information. The ability to recall and locate the virtual objects was the same in men and women, but there were differences in the speed of exploration of the environment in the learning phase. Men spent more time than women.

One possible interpretation of the similar results between men and women in the ability to locate the virtual objects may be related to the spatial strategy used. This task might require the use of a route strategy. Several studies have shown that women are prone to use a route strategy, and men seem to prefer a survey strategy (Lawton, 1994, 1996; Lawton and Kallai, 2002). In this study, we did not specifically ask the participants about the strategy they had used in the AR task. However, while the participants were performing the AR task, we took notes of the comments that were related to the spatial strategy used. Most of these comments were related to the use of a route-based strategy, probably because distal cues are used more unconsciously and, therefore, they are more difficult to report verbally. However, it should also been taken into consideration that spatial strategies are cumulative and, therefore, survey representation is characterized by the properties of landmark, route, and survey representation together (Lugli et al., 2017). If both men and women used this type of route orientation, it is not surprising that both sexes performed similarly on the task. In fact, no gender difference emerged when only landmark cues were available in the task, so a survey strategy was not possible (Sandstrom et al., 1998; Saucier et al., 2002).

Additionally, it has been described that familiarity with the environment can influence participants’ performance in a real environmental spatial learning task, improving skills (Nori and Piccardi, 2011; Nori et al., 2018). In fact, familiarity with the environment allows more successful navigation in people with a poorer navigation style (Piccardi et al., 2011a), and the effect is more evident when, as in our experiment, participants move around freely in a real environment. So, we cannot discard that the lack of gender differences observed in our experiment might be due to the fact that all the participants were familiarized with the building. This variable could have allowed the participants with a more deficient cognitive style (route) to perform the AR spatial task more accurately.

Another possible explanation is the difficulty of the task. It has been suggested that gender differences emerge when the task has an optimum level of difficulty. If the level was very low or the participants were allowed to repeat the task as many times as needed, or to take their time to perform it, both sexes performed similarly (Piccardi et al., 2011b; León et al., 2016). Our task is partially in line with these characteristics because the participants did not have a maximum time to perform the task. However, the difficulty of the task was not low. As stated above, no participant was able to locate all of the objects. We suggest that suppressing the time pressure can significantly contribute to eliminate gender differences.

Surprisingly, we observed that men spent more time learning the location of the objects. Although these results may seem to go against the previous literature (Piccardi et al., 2011b; Nori et al., 2018), we hypothesized that the fact that men took more time in the learning phase was not related to spatial orientation, but rather to their interest in the technology we used in this experiment. We observed that men were more enthusiastic about the AR app than women, paying more attention to how the AR app works and taking more time to observe the AR objects in detail, from a closer distance. In fact, differences were observed only in the learning phase, that is, when participants interacted with the AR app for the first time. No gender differences were observed in the testing phase. Our hypothesis might be supported by a recent study using a mobile AR game. In this article, researchers observed that men were more interested than women in a mobile AR game (Delello et al., 2018).

Our results show no significant gender differences in the map-pointing task, either in the percentage of correctly positioned objects or in the time needed to complete the task. In this task, participants had to point to the virtual objects they had previously seen in the AR task on a map. Only a few landmarks were present (i.e., the stairs; see Figure 6), so their success was dependent on the creation and use of a cognitive map. More specifically, the participants needed to transform the route representation of the environment (based on spatial cues) into an allocentric representation of the environment (Wolbers and Wiener, 2014). Thus, individuals who can restructure route information into allocentric information might perform this task better. Taking into consideration the results obtained in these two orientation tasks (the AR and map-pointing task), we hypothesize that the men and women who participated in our study did not differ in the spatial strategy used to orientate themselves in these tasks. The participants were volunteers who wanted to test their spatial abilities. Therefore, it is possible that those potential participants who were not self-confident about their spatial orientation did not want to participate in the study. Therefore, our results could be in line with other studies, in which gender differences in spatial orientation were not observed in participants with high spatial abilities and a lot of experience (Verde et al., 2013, 2015). Similarly, Boccia et al. (2017) demonstrated that women and men did not differ in their performance on spatial memory tasks, when they had the same field-dependent/independent cognitive style (referring to the way in which people organize environmental information), as they adopt similar strategies.

Another aspect that supports our hypothesis is the lack of differences between men and women in the self-reported spatial strategies. Despite the fact that most of the studies reported differences between men and women in the spatial strategy preferred (Lawton, 1994, 1996; Lawton and Kallai, 2002), Castelli et al. (2008) also observed that both sexes reported similar importance of the route and survey strategies for orientation. However, the self-reported questionnaires about orientation strategies measure the participants’ own spatial perceptions. The self-reported gender differences in spatial orientation tend to parallel people’s performance on spatial tasks (Montello and Pick, 1993).

Our data are not in line with previous investigations using self-reported questionnaires in which men and women differed in their strategy of preference (Lawton, 1994, 1996; Lawton and Kallai, 2002). However, the explanation of why women prefer to use a route strategy instead of a survey strategy was related to their lower wayfinding experience in childhood compared to men (Lawton and Kallai, 2002; Schug, 2016).The fact that women had less freedom to explore the environment during childhood has stunted the development of their spatial skills, leading to higher anxiety during spatial orientation tasks (Lawton and Kallai, 2002; Schug, 2016). However, similarly to the results of Castelli et al. (2008), we did not find gender differences in the degree of importance of the three different wayfinding strategies assessed. In addition, the men and women of our study had the same wayfinding experience during childhood. This could explain their similar level of wayfinding anxiety and their similar levels of preference for spatial strategies.

Whether there is a relation between spatial orientation ability (assessed by large-scale spatial tasks such as the AR task) and the spatial ability assessed by paper-and-pencil psychometric tests (small-scale tasks) is still under debate. Despite the fact that the literature provides considerable evidence that processing spatial information in small-scale spatial tasks (i.e., mental rotation, perspective taking) and in large-scale spatial tasks involves different brain mechanisms and regions (Philbeck et al., 2000; Kosslyn and Thompson, 2003), some studies have argued that perspective-taking ability is related to the performance on large-scale navigation tasks (Kozhevnikov et al., 2006; Pazzaglia and Taylor, 2007). In addition, gender differences have been extensively found in paper-and-pencil tests, such as mental rotation tests (Linn and Petersen, 1985; Voyer et al., 2006; Jansen and Heil, 2009). In our study, gender differences emerged in the performance of the PTSOT, a psychometric test for assessing gender differences in perspective-taking and in the ability to make egocentric spatial transformations (Kozhevnikov and Hegarty, 2001; Hegarty and Waller, 2004). Our results support previous studies in which women had a poorer performance on the PTSOT than men (Meneghetti et al., 2012; Zancada-Menendez et al., 2016). As in the case of our work, these studies considered the degrees of error for establishing comparisons between sexes. Other studies that considered the number of correct answers found no gender differences (Hegarty et al., 2006; Iwanowska and Voyer, 2013; Zancada-Menendez et al., 2016).

The relation among the studied variables was also investigated. As expected, the number of objects that were correctly located in the AR task correlated positively with the percentage of correct objects pointed to in the map task. This could indicate that the route representation of the environment (based on spatial cues) needs to be transformed into an allocentric representation of the environment (Wolbers and Wiener, 2014). In fact, these two measures of performance correlated negatively with the percentage of objects omitted during the recall. The more objects located correctly, the fewer the objects omitted in the recall.

On another hand, in our study, we observed a lack of correlations among the PTSOT, the AR, and map-pointing tasks. Our results are in accordance with previous studies in which the performance on spatial large-scale environmental tasks did not correlate with the performance on spatial psychometric tests (Mitolo et al., 2015). In addition, information processing and the brain regions involved were different between small-scale and large-scale spatial tasks (Philbeck et al., 2000; Kosslyn and Thompson, 2003).

The correlation analysis showed a relation between anxiety and spatial orientation. As expected, high levels of trait anxiety were related to high levels of fear of getting lost. Another study found a similar result (Lawton and Kallai, 2002). People who are more anxious in general situations may also experience more anxiety when they have to perform spatial tasks or they may even be more afraid of getting lost.

It is noteworthy that trait anxiety and the time spent by the participants to complete the learning phase of the AR task correlated positively. Trait anxiety influences cognitive domains such as attention and concentration (Bishop, 2009; Vytal et al., 2013). Anxiety could impair these processes and negatively affect performance. However, in accordance with our data, highly anxious individuals could perform well by increasing their effort, that is, spending more time on the task (Eysenck and Calvo, 1992). Similarly, withdrawal behaviors in children were related to an increase in exploratory behaviors in a virtual spatial memory task, but without effects on spatial learning (Rodriguez-Andres et al., 2018).

Interestingly, we found a significantly negative correlation between wayfinding anxiety and the orientation strategy, regardless of gender. This result supports previous results that showed a low preference for the orientation strategy in individuals with higher levels of wayfinding anxiety (Lawton and Kallai, 2002).Taken together, these results are in line with previous research showing that emotional factors are relevant for the study of individual differences in spatial orientation (Lawton and Kallai, 2002; Walkowiak et al., 2015; Schug, 2016; Pazzaglia et al., 2018).

The present research has some limitations. First, it would have been desirable to increase the sample size. Second, the way in which participants were recruited could have had a deterrent effect on participants with low self-confidence in spatial orientation. However, as discussed above, the lack of gender differences in the AR and the map tasks could be due to familiarity with the building, or to the lack of differences observed between men and women in self-reported strategies, wayfinding anxiety, and wayfinding experience, all of which are related to performance on spatial tasks.

## Conclusion

For the first time, we have used an AR app to test spatial memory for the location of virtual objects that were shown when the person navigated different floors of a building. We have also related this spatial memory performance to spatial factors and anxiety levels. For the first time, we can say that the AR app used in this experiment is a useful technology for assessing spatial orientation in complex, real-world environments. We found that gender did not affect the performance of either the complex real-world spatial task or the map-pointing task in men and women with similar wayfinding experience, preference for spatial strategies, and levels of anxiety. Gender dimorphism appeared in our paper-and-pencil test of spatial orientation, suggesting that the real-world spatial task and the map-pointing task assess spatial competences that are different from those assessed in the paper-and-pencil test. On another hand, anxiety was related to individual differences in the preference for an orientation strategy and the time taken to complete the learning phase of the AR task. Our results highlight the importance of anxiety in spatial tasks. However, more research is needed to further investigate how other emotional factors such as personality or motivational aspects may influence spatial orientation. In particular and considering the possibility that the AR task offers, a new research goal could be to study the variation in levels of state anxiety and related performance outcomes using the AR task. In addition, the effect of age could be considered.

## Data Availability

The datasets for this manuscript are not publicly available because the raw data supporting the conclusions of this manuscript will be made available by the authors, without undue reservation, to any qualified researcher. Requests to access the datasets should be directed to MM-L, mmendez@unizar.es.

## Author Contributions

CF, FM-M, M-CJ, and MM-L conceived, designed and developed the app. CF, M-CJ, and MM-L conceived, designed and performed the experiments. CF and MM-L analyzed the data. CF, FM-M, M-CJ, and MM-L interpreted the data and drafted the manuscript.

## Conflict of Interest Statement

The authors declare that the research was conducted in the absence of any commercial or financial relationships that could be construed as a potential conflict of interest.
